# Diversity, Ecology and Herbivory of Hairstreak Butterflies (Theclinae) Associated with the Velvet Tree, *Miconia calvescens* in Costa Rica

**DOI:** 10.1673/031.010.20901

**Published:** 2010-12-23

**Authors:** F. R. Badenes-Péérez, M. A. Alfaro-Alpíízar, M. T. Johnson

**Affiliations:** ^1^ Pacific Cooperative Studies Unit, University of Hawaii at Manoa, 3190 Maile Way, Honolulu, HI 96822; ^2^ Centro de Ciencias Medioambientales, Instituto de Ciencias Agrarias, CSIC, 28006 Madrid, Spain; ^3^ Escuela de Biologíía, Universidad de Costa Rica, San Pedro de Montes de Oca, San Jose, Costa Rica; ^4^ Institute of Pacific Islands Forestry, USDA Forest Service, Pacific Southwest Research Station, Volcano, HI 96785, USA

**Keywords:** biodiversity, butterflies, *Erora opisena*, hairstreak, inflorescence, Lepidoptera, Lycaenidae, *Parrhasius polibetes*, *Temecla paron*, Theclinae

## Abstract

Larvae of three species of hairstreak butterflies in the subfamily Theclinae (Lepidoptera: Lycaenidae) were found feeding on developing inflorescences, flower buds, and immature fruits of the velvet tree, *Miconia calvescens* DC. (Myrtales: Melastomataceae) in Costa Rica. *Erora opisena* (Druce), *Parrhasius polibetes* (Cramer), and *Temecla paron* (Godman and Salvin) were studied in association with *M. calvescens*, an uncommon tree in its natural range in the neotropics and a target for biocontrol as an invader in Pacific islands. Host plant use by the three theclines was similar, with eggs being laid on inflorescences and cryptic larvae remaining there throughout development. Feeding damage by *E. opisena* was most abundant in pre-flowering *M. calvescens*, when 23% of inflorescences showed feeding damage characteristic of this species. Feeding damage by *T. paron* peaked at flowering, when 30% of inflorescences were affected. At field sites, *E. opisena* and *T. paron* damaged an average of 26 and 18% of each attacked inflorescence, respectively. In cage experiments, individual third- and fourth-instar larvae of *E. opisena* damaged an average of 24 and 21% of an inflorescence before pupating, respectively. This study provides the first host plant record for *E. opisena* and *T. paron*, the first record of *P. polibetes* feeding on Melastomataceae, and the first records of *E. opisena* and *T. paron* presence in Costa Rica.

## Introduction

The velvet tree, *Miconia calvescens* DC. (Myrtales: Melastomataceae), is a small tree native to Central and South America, although it is also present as an invasive species in Hawaii and other Pacific islands (Denslow et al. 1990; Medeiros et al. 1997; Meyer and Florence 1996). The success of *M. calvescens* as an invasive plant is largely due to its prolific production of bird-dispersed fruit, with one mature tree flowering up to three times per year and bearing up to 220 inflorescences that can produce more than 200 fruits, each with 25–200 seeds per fruit (Badenes-Perez and Johnson 2007a; Medeiros et al. 1997; Meyer 1998). Classical biological control via the introduction of natural enemies from the neotropics is considered an essential tool for long-term management of *M. calvescens* (Denslow and Johnson 2006; Kaiser 2006; Smith 2002). Reducing reproduction of *M. calvescens* with insects that feed on inflorescences and fruit may prove beneficial by slowing its spread into new areas.

In its native habitat, inflorescences and fruits of *M. calvescens* are used by a variety of insect herbivores (Badenes-Perez et al. 2008; Badenes-Perez and Johnson 2007a; Chacóón 2007) among which some are theclines (Lepidoptera: Lycaenidae: Theclinae). Lycaenidae is the most species-rich family of butterflies, accounting for about one third of all true butterflies (Papilionoidea) (Robbins 1982). Lycaenidae species are distributed throughout the world, with about 25% of the species located in the neotropics (DeVries 1997). Like most lycaenids, larvae of the subfamily Theclinae are cryptic and feed on floral buds, flowers, and fruits (Downey 1962; Fiedler 1995; Fiedler 1997). Tropical theclines are often beautiful hairstreaks with wings of iridescent blue color above, caused by reflected light from the their wing scales (D'Abrera 1995; Lamas 2004; Robbins 2004). In the neotropics, many species in this group of butterflies are considered rare and there is a lack of information on their distribution and ecology, which is necessary to ensure their conservation.

Three different field sites in Costa Rica were surveyed to study the presence of theclines that use *M. calvescens* as a host and to study their abundance, ecology, and herbivory on *M. calvescens* inflorescences. Because of different climatic conditions, habitat fragmentation, and physical separation between field sites (>60 km), different theclines could occur in each field site. Besides providing information on rare butterflies for which little is known, this is the first study of these inflorescence-feeding species as potential biological control agents of *M. calvescens.*

## Materials and Methods

Thecline caterpillars were collected at three field sites in central and eastern Costa Rica within 70 km of San Jose. At each site, 50–200 *M. calvescens* plants had been planted between 2003 and 2005 in a cleared plot about 300–1000 m2 to increase the abundance of this normally uncommon plant, and raise the likelihood of attracting potentially host-specific herbivores. At the time of this study plants ranged from immature trees about 1 m high to mature trees about 4 m high. At two sites there were also natural populations of *M. calvescens* present, consisting of 10–20 mature trees up to 8 m in height within several contiguous hectares of lightly disturbed native secondary rainforest. At the Cariblanco site (10°° 18′? 59″? N and 84°° 11′? 00″? W, 986 m above sea level) conditions were very wet (>7000 mm annual rainfall) with no dry season. The Veréé site (09°° 40′? 00″? N and 83°° 31′? 40″? W, 1200 m above sea level) was less wet (3000 mm annual rainfall) with a 1–2 month dry season. The third site at Sabanilla de Montes de Oca (09°° 56′? 48″? N and 84°° 02′? 45″? W, 1200 m above sea level) was in highly disturbed suburban San Jose, far from native forest areas and natural populations of *M. calvescens.* Here conditions were much drier (1800 mm annual rainfall) with a relatively severe 3–4 month dry season.

Laboratory studies were conducted at the Department of Biology of the University of Costa Rica in San Pedro de Montes de Oca. During the period of this study, October 2006 to February 2007, the laboratory experienced a photoperiod of approximately 11–12:13–12 L:D, 22 ±± 3°° C and 68 ±± 5% relative humidity.

### Abundance and phenology of theclines on *M. calvescens*

Theclines found feeding on *M. calvescens* were recorded over the course of one flowering and fruiting season at each of the three field sites. At the Cariblanco and Veréé sites, larval densities were surveyed by inspecting at least five *M. calvescens* inflorescences per tree in 5–10 trees on six dates from October or November 2006 to March 2007 (a minimum of 50 inflorescences examined at each site on each survey date). Because the cryptic larvae were difficult to observe in the field, 25 of the *M. calvescens* inflorescences examined in the field were brought to the laboratory for careful inspection. Inflorescences were held in plastic containers (45 ×× 30 ×× 15 cm) and were inspected 3 times (every 2 days) before being discarded. At the Sabanilla site, only 3 reproductive *M. calvescens* were present, and only 5 inflorescences were inspected 4 times in total between January and April 2007. On each survey date, the phenology of *M. calvescens* was assessed in 10 randomly selected inflorescences per tree (or in all if the tree had less than ten inflorescences) by rating the present inflorescences as predominantly floral buds, opened flowers, immature/green fruit, or mature/red-purple fruit.

### Biology of immature theclines

Inflorescences collected in the field and brought to the laboratory (see above) were inspected for eggs and larvae. Larvae found in the inflorescences brought to the laboratory were reared individually either on *M. calvescens* inflorescences kept with humid vermiculite in plastic pots (6 cm diameter by 10 cm height) and/or on small *M. calvescens* leaves (12–15 cm long) folded (with adaxial side exposed to the larvae) and kept in Petri dishes (9 cm diameter by 1.4 cm height). Larvae in Petri dishes were transferred to a fresh leaf every 48 h until they pupated. Voucher specimens of the reared adult Theclinae were deposited in the Zoology Museum of the Department of Biology at the University of Costa Rica.

### Thecline herbivory on *M. calvescens*

During field surveys, inflorescences were also inspected for signs of feeding damage by theclines which included holes in floral primordia, sections of flowers cut from the base, and hollowed green fruits. Data were recorded as number of inflorescences with damage per tree. To assess thecline feeding in greater detail, 35 damaged inflorescences (5 inflorescenes for each 7 *M. calvescens* plants) were collected and brought to the laboratory for examination on 30 November 2006 (from Cariblanco) and on 4 December 2006 (from Veréé). Numbers of flowers damaged and not damaged were counted for each inflorescence.

To quantify damage per individual, larvae of is. *opisena* collected at the field site in Cariblanco were placed on caged inflorescences of a mature *M. calvescens* planted on the main campus of the University of Costa Rica in San Pedro de Montes de Oca. Ten newly molted larvae (5 third instars and 5 fourth instars) were placed individually on 10 inflorescences caged with aluminium window screening to prevent parasitism/predation. Feeding damage by these late instars, expected to have greater impact than feeding by early instars, was assessed after pupation by counting the number of flowers fed upon and by calculating the percentage of the inflorescence damaged by the larvae. Racemes in each inflorescence were counted and damage to distal versus basal racemes was recorded to evaluate the pattern of damage within the inflorescence. At the site of this experiment, *M. calvescens* experienced little natural herbivory and environmental conditions were similar to the site in Sabanilla (about 1.5 km away).

### Statistical analysis

Larval densities and damage in inflorescences were evaluated using analysis of variance (ANOVA) with the PROC GLM procedure of SAS (2004). When treatment differences were indicated by a significant F-test, means were separated by Fisher's Protected least significant difference (SAS 2004). Data comparing feeding damage between the distal versus basal parts of the inflorescence were analyzed using a paired t-test analysis of variance (ANOVA) with the PROC TTEST procedure of SAS (2004). In order to normalize the residuals, an LN (x+1) function was used to transform data. Although tests of significance for these analyses were based on the transformed data, only untransformed data are presented.

## Results

### Abundance and phenology of theclines on *M. calvescens*

A different thecline was collected at each of the three field sites. *Erora opisena* (Druce), *Temecla paron* (Godman and Salvin), and *Parrhasius polibetes* (Cramer) were found associated with *M. calvescens* in Cariblanco, Veréé, and Sabanilla de Montes de Oca, respectively. Overall the numbers of caterpillars found were low, indicating the rarity of these species, at least on *M. calvescens.* In the inflorescences inspected in the field and in the laboratory, the total number of *E. opisena* larvae found were 19, 1, 1,0, and 0 for the sampling conducted in October (when inflorescences were predominantly preflowering floral buds), November (open flowers), January (immature fruit), and February and March (mature fruit), respectively. The total number of *T. paron* larvae found were 4, 1, 0, 0, and 0 for the sampling conducted in November (floral buds), early December (open flowers), late December (immature fruit), and February and March (mature fruit), respectively. Only two larvae of *P. polibetes* were found in Sabanilla, one in January (floral buds) and one in February (open flowers). Flowering started approximately three weeks earlier in Cariblanco than in Veréé. Flowering started in Sabanilla de Montes de Oca approximately three months later than in Cariblanco.

### Biology of immature theclines

Eggs of *E. opisena* were seen in only 4 of all inflorescences inspected: 3 inflorescences collected in September with 2, 3, and 6 eggs each; and 1 inflorescence collected in October with 2 eggs. Eggs were whitish and hemispherical with a rough surface, and measured approximately 0.55 mm in diameter. Eggs were found on the bracts of inflorescences during the floral bud stage. Larvae of *E. opisena* were found in numbers ranging from 1 to 3 larvae per inflorescence (up to 6 larvae per tree). Eggs of *T. paron* were found in the field only once (2 eggs in one inflorescence in the floral bud stage in November) and were similar to those of *E. opisena.* No more than one *T. paron* larva was found per inflorescence.

In the laboratory, larvae of *E. opisena* and *T. paron* ate their cast skins after each molt. The number of instars based on head capsule measurements was determined only for *E. opisena* (four instars). Of all the larvae collected from the field (21 *E. opisena* and 5 *T. paron*), only 1 *E. opisena* and 1 *T. paron* were parasitized by an undetermined parasitoid and a *Conura* sp. (Hymenoptera: Chalcididae), respectively.

Inspection of *M. calvescens* leaves and stems around inflorescences with damage from *E. opisena* and *T. paron* never revealed pupae, indicating that larvae may descend from plants to pupate in the soil or surface litter. In the laboratory, both *E. opisena* and *T. paron* pupated in Petri dishes between folded *M. calvescens* leaves or descended from inflorescences to pupate near the base of the plastic pot. In laboratory conditions, adults emerged from pupae after 3–5 days and lived 5–8 days. In the field, adults of *E. opisena* were observed only once, flying around inflorescences of *M. calvescens* in Cariblanco on 30 November 2006 at 11:00 am.

**Figure 1.**
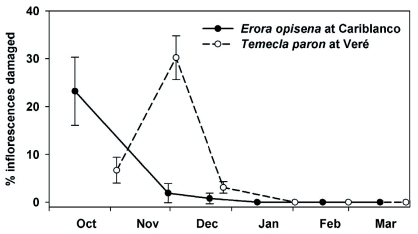
Average percentage ±±SE of *Miconia calvescens* inflorescences damaged by *Erora opisena* and *Temecla paron* at Cariblanco and Veréé field sites, respectively. Surveys conducted between October 2006 and March 2007 as inflorescences matured through four distinct phenological stages (floral buds, open flowers, immature fruit, and mature fruit). High quality figures are available online.

### Thecline herbivory on *M. calvescens*

The percentage of *M. calvescens* inflorescences showing damaged by *E. opisena* in the field changed through the sampling period (*F* = 17.11, df = 5, 24, *P* < 0.001) (Figure 1). The percentage of inflorescences damaged by *E. opisena* was highest during the sampling conducted during the floral bud stage (14 October), with 23.2% of all inflorescences sampled showing characteristic damage. The percentage of *M. calvescens* inflorescences damaged by *T. paron* in the field also changed through the sampling period (*F* = 9.18, df = 5, 24, *P* < 0.001), with more inflorescences damaged by *T. paron* at the floral bud (4 November) and open flowers (4 December) stages and with 6.7 and 30.2% of all inflorescences sampled showing damage, respectively (Figure 1). Within thecline-damaged inflorescences, an average (±± SE) of 26.0 (±± 5.2) and 17.7% (±± 3.6) of each inflorescence showed damage by *E. opisena* at Cariblanco and *T. paron* at Veréé, respectively.

In the field experiment at the University of Costa Rica, individual third instars of *E. opisena* damaged 97 ±± 27 flowers (23.9% of all flowers in the inflorescences sampled) in their development to pupation. Individual fourth instars of *E. opisena* damaged 137 ±± 27 flowers (21.6% of all flowers in the inflorescences sampled) while completing their development to the pupal stage. Damage caused by all larvae appeared to be uniformly distributed throughout the inflorescence, and there were no differences between distal and basal portions (*t* = 0.255; df = 1,9; *P* = 0.804).

## Discussion

*Miconia calvescens* is the first host plant ever reported for *E. opisena* and *T. paron*, two rare species of butterflies. This is also the first record of *E. opisena* and *T. paron* in Costa Rica. *Erora opisena* is considered a rare cloud forest species (Robbins RK, personal communication) and has been previously collected only in Mexico and Colombia (D'Abrera 1995; Robbins 2004). Although the host-specificity of *E. opisena* is not known, other *Erora* spp. that have been reared were polyphagous (Robbins RK, unpublished data). *Temecla paron* has been collected in Mexico and Guatemala (D'Abrera 1995; Godman and Salvin 1887b; Lamas 2004; Robbins 2004). No *Temecla* spp. have been previously reared, and all *Temecla* spp. are rare to very rare in museum collections (Robbins RK, personal communication). Unlike *E. opisena* and *T paron, P. polibetes* is a more common and better known species and its larvae have been found feeding on Euphorbiaceae (Zikan 1956), Fabaceae (D'Araujo e Silva et al. 1968), Malpighiaceae (http://janzen.sas.upenn.edu/), and Vochysiaceae (Diniz and Morais 2002) from Mexico to Brazil (D'Abrera 1995; Godman and Salvin 1887a; Lamas 2004; Robbins 2004). This study is the first record of *P. polibetes* on Melastomataceae. *Parrhasius polibetes* is a larger thecline than *E. opisena* and *T. paron*, with the larvae growing to about twice the size of *E. opisena* and *T. paron* on *M. calvescens.* The number of larval instars of *T. paron* and *P. polibetes* was unable to be determined, but with the exceptions of one gymnosperm-feeding species with six instars (Ballmer and Pratt 1988) and one detritivore species with five (Duarte et al. 2005), all studied Lycaenidae have four instars (Robbins, pers. comm.).

The measurements in this study showed that larvae of these theclines feed extensively on *M. calvescens* inflorescences, particularly in the early stages of inflorescence development. Further studies are needed to better understand host specificity in these butterflies. At field sites in Costa Rica, *M. calvescens* lacks inflorescences for several months each year (Chacóón 2007), which suggests that these herbivores probably rely on other hosts during that time. Larvae of these theclines were able to develop on *M. calvescens* leaves in the laboratory and this could allow them to temporarily survive without inflorescences, but this was never observed in the field during extensive inspections of plants. Larval food plants of neotropical Lycaenidae are poorly known in general, but approximately 95% of neotropical Lycaenidae (including the genera *Erora, Temecla*, and *Parrhasius)* belong to the tribe Eumaeini (Lamas 2004; Robbins 2004), which is considered polyphagous as a whole (Chew and Robbins 1984). Two theclines from Mexico, *Strymon bazochii* (Godart) and *Tmolus echion* (L.), were released as biological control agents of the invasive weed *Lantana camara* L. (Verbenaceae) in Hawaii in 1902, but have since been recorded eating flowers of species belonging to many plant families (Perkins and Swezey 1924; Robbins and Aiello 1982; Zimmerman 1958) and not having any significant impact on the weed (Julien and Griffiths 1998).

Although parasitism of *E. opisena* and *T. paron* in their native habitat was low and the cryptic appearance of these species on *M. calvescens* may help them avoid some natural enemies, the probability of biotic interference affecting these species in Hawaii is high because by feeding externally they would be exposed to a variety of generalist enemies of Lepidoptera (Davis et al. 1992; Henneman and Memmott 2001). Compared to other insect herbivores associated with *M. calvescens*, these theclines show low potential as biological control agents (Badenes-Perez et al. 2008; Badenes-Perez and Johnson 2007a; Badenes-Perez and Johnson 2007b; Badenes-Perez and Johnson 2008).

*Miconia calvescens* is considered uncommon as a mature tree in its native habitat, and is instead restricted to secondary vegetation, forest edges, and forest gaps (Denslow et al. 1990; Ellison et al. 1993; Meyer 1998). Habitat loss and fragmentation likely contribute further to the uncommon and scattered presence of *M. calvescens* in its native range. If *E. opisena* and *T. paron* specialize on a few plants, *M. calvescens* could play an important role in the survival and conservation of these rare butterflies in Costa Rica, at least at a local and seasonal level.
